# The Socioeconomic Impact of Diseases of Working Equids in Low and Middle-Income Countries: A Critical Review

**DOI:** 10.3390/ani13243865

**Published:** 2023-12-15

**Authors:** Marta Bonsi, Neil E. Anderson, Gemma Carder

**Affiliations:** 1 The Royal (Dick) School of Veterinary Studies and The Roslin Institute, University of Edinburgh, Midlothian EH25 9RG, UK; 2 Brooke, Action for Working Horses and Donkeys, 2nd Floor, The Hallmark Building, 52-56 Leadenhall Street, London EC3A 2BJ, UK

**Keywords:** working equids, ‘One Health’, animal health economics, animal welfare, livelihoods, low- and middle-income countries, critical review

## Abstract

**Simple Summary:**

Working equids represent a source of livelihood for vulnerable communities in low and middle-income countries. This has been widely demonstrated by research conducted in various contexts, which is available to policymakers. Nevertheless, the important social and economic role of working equids is overlooked and they are excluded by governments and international organisations from animal health policies and interventions, with detrimental effects on animal welfare, human wellbeing and livelihoods. By reviewing the literature available on the subject, this study aims to investigate the effects of diseases of working equids on the economic and social assets of their owners. Results show that working equid diseases severely damage livelihoods, human health and social assets. This study explores the relationship between working equid health, livelihoods and human health according to the ‘One Health’ approach that enables us to evaluate the implications of a particular problem on human, animal and environmental health. Through the ‘One Health’ approach, this study will serve as a resource to sensitise policymakers on the need to develop policies and interventions aimed at protecting the health and welfare of working equids and, consequently, the health and livelihoods of their owners. It is hoped that the ‘One Health’ approach applied within this study will provide an opportunity to also reach those policymakers who have not yet recognised the multiple implications of working equid health on human health, wellbeing and livelihoods.

**Abstract:**

Working equids provide a crucial contribution to the livelihoods and food security of communities in low- and middle-income countries (LMICs). Nevertheless, they are a neglected category within animal health policies and interventions of governmental and non-governmental institutions. This critical review aims to assess the socioeconomic impact of diseases of working equids in LMICs. By highlighting the implications of diseases on working equid welfare, human wellbeing and livelihoods, this review seeks to sensitise policymakers within governments and international organisations to develop policies and interventions aimed at protecting the health of working equids and, consequently, the health and livelihoods of their dependent communities. Twenty relevant publications were identified through the search of five databases (CAB Abstracts, Web of Science Core Collection, BIOSIS, EMBASE and Scopus), backward citation searching and screening of indexes of proceedings and Special Issues retrieved from the database search. The review findings show that diseases of working equids have detrimental socioeconomic effects. However, this subject is under-researched and restricted to few diseases and geographical settings. Considering the complexity of the issue, this review demonstrates that the ‘One Health’ approach represents an opportunity to clarify the link between equid health, human wellbeing and livelihoods, facilitating the translation of research into policy.

## 1. Introduction

Working equids are horses, donkeys and mules providing draught power and transportation for income-generating activities and domestic purposes [1,2]. There are around 116 million working equids worldwide, representing 92% of the global equid population. Working equids are a source of livelihood for 600 million people in low- and middle-income countries (LMICs) [3,4]. The socioeconomic contribution of working equids to the livelihoods of communities in LMICs has been researched in East Africa [5], West Africa [6], Central Africa [7], South Africa [8], South Asia [9] and Central America [10]. These studies show that working equids are engaged in a great variety of activities with multiple benefits for their owners. Working equids are a direct and indirect source of income for urban and rural communities, contributing to food security and enabling households to cover their basic needs [1]. Income is generated directly by the transportation of commodities and people, by hiring the animals out and selling their products such as offspring and manure. Indirect income originates by supporting other income-generating activities like trading, agriculture and livestock rearing [1,11,12]. Working equids contribute to national economies by working in the construction, public transport, mining, tourism, and agricultural sectors [1,13]. Moreover, they provide essential services to communities such as waste management [14] and filling infrastructural gaps, for example water distribution in the absence of public water systems [15]. Working equids have crucial social roles as they are loaned to community members, reinforcing relationships and social status. They are also engaged during religious and traditional events. Working equids are used for domestic tasks like water and firewood transportation and for carrying the sick to hospitals and children to schools. By covering these tasks, working equids allow families to save on transportation and labour costs and they reduce the work burden on women [1,9,16]. In pastoral settings, thanks to working equids, entire families can move through their migration routes [13]. It should not be underestimated that the activities carried out by working equids are at low-carbon emissions [1]. Lastly, working equids can be sold to generate cash in difficult times, strengthening household resilience [12,16]. By exercising all of these functions, working equids contribute to the achievement of several of the sustainable development goals (SDGs) in terms of food security, gender equality, access to education, creation of employment, water provision, poverty reduction, clean energy, and human health promotion [17].

Regardless of these multiple socioeconomic roles, there is a widespread lack of attention to working equids’ health and welfare. This is caused by a combination of factors, including lack of owners’ knowledge and economic means, the unavailability of appropriate and affordable drugs and lack of veterinary services. These factors are all directly or indirectly attributable to the absence of working equids from animal health policies of governments and international organisations [12,18,19]. Diseases of working equids are linked to their working conditions and environment. Wounds and lameness are caused by inappropriate harness and cart, overloading, overworking and road accidents [20], while infectious diseases, including zoonoses, spread easily in overcrowded equid stations [18,21]. It is not uncommon to observe malnourished animals due to chronic diseases or lack of food. Overworked animals become dehydrated and exhausted and suffer from stress as they cannot express their natural behaviour [22]. If untreated, these conditions are detrimental to animal welfare, frequently leading to death. Moreover, they have a negative impact on the productivity and income-generating capacity of equids, causing severe damage to households that rely on a single animal as a source of livelihood [9,12]. Many working equid diseases could be prevented by improving owners’ knowledge on biosecurity, sustainable harness and basic animal care and welfare. This could be achieved by providing veterinary services, including extension services for the provision of treatment, immunisation campaigns and owner education. These activities are currently implemented by a limited number of service-providers in LMICs [22,23] such as a few international charities, some initiatives run by the Western diaspora in touristic locations and by some local organisations. With exceptions from the areas where these institutions operate, there are several gaps in geographical coverage, and so, the health problems of working equids remain unaddressed, especially in remote locations [16,24] and war-affected countries. In these contexts, government clinics and extension agents rarely address the health needs of working equids as they focus mostly on ruminants [1,9].

Regardless of the high global population of working equids and their support to people’s livelihoods, working equids are excluded from animal health and welfare policies of governmental and non-governmental institutions. Consequently, they are not considered within disease surveillance and control programmes implemented by governments and international organisations in LMICs, ignoring that many of their diseases are notifiable to the World Organisation of Animal Health (WOAH) [25,26]. This is also reflected in the absence of working equid-related topics within training for extension agents, veterinarians and stakeholders involved in animal health programmes in LMICs, resulting in a widespread lack of knowledge on disease treatment and welfare needs that makes working equids even more neglected [1]. An improved knowledge among policymakers on the epidemiology and socioeconomic impact of diseases of working equids under a transdisciplinary perspective may promote the understanding of the relationship between working equid health, human health and livelihoods and, consequently, the development of policies aimed at protecting health and welfare of working equids [9]. This transdisciplinary viewpoint could be provided through the ‘One Health’ approach that considers health under a holistic perspective and it is aimed at improving human, animal, and ecosystem health [27]. The ‘One Health’ concept was developed in response to the pandemics that have occurred in the past 20 years. It is based on the collaboration of experts from different disciplines including both natural and social sciences [27] and on the contribution of non-academic knowledge such as indigenous knowledge [28]. These multiple combinations of knowledge explain the transdisciplinary nature of the ‘One Health’ method. Although equids are a relevant part of ‘One Health’ because of equine zoonoses, the human-equid relationship, their socioeconomic role in LMICs and their impacts on the ecosystem [29], there is a very limited amount of research targeting working equids that refers to the ‘One Health’ approach [18,30,31,32].

The objective of this study is to review the existing knowledge on the socioeconomic impact of diseases of working equids in LMICs and to identify research gaps on the topic. A desk-based study was initially conducted and, due to the importance of the female-gender perspective on the subject, the authors decided that this specific aspect of the research deserved to be widened in a separate article. A scoping review on the female-gender perspective of health problems of working equids in low- and middle-income countries was recently published by the authors [33]. By highlighting disease effects under a ‘One Health’ viewpoint, and in particular their implications on human health, wellbeing, and livelihoods, this review is intended to serve as a resource to sensitise policymakers within governments and international organisations in LMICs on the importance of developing policies and animal health programmes aimed at protecting the health of working equids and, consequently, the health and livelihoods of the communities depending on them. Through the ‘One Health’ lens, the link between equid, human and ecosystem health can be effectively uncovered, representing an opportunity to reach those policymakers who have not yet recognised the holistic dimension of working equid health.

## 2. Materials and Methods

### 2.1. Study Design

Since literature reviews summarise evidence-based knowledge and highlight potential areas of research, they have increased in popularity among decisionmakers in various fields [34]. Based upon this, a critical review of the literature on the socioeconomic impact of diseases on working equids in LMICs was conducted with the aim to promote change, in particular by influencing policymakers, in line with a pragmatism methodology [35]. The critical review method was selected so that the discussion could focus on the relevance and potential impact of existing knowledge and on how this subject could be further developed [34,36]. Systematic reviews are considered the gold standard for informing policy because of their objectivity, replicability and exhaustiveness. Nevertheless, the partial application of guidelines for systematic reviews, for instance, in terms of structured research methods, represents an advantage to increase the robustness of other review types [37]. For this reason, some elements of the preferred reporting items for systematic reviews and meta-analysis for literature searches (PRISMA-S) guidelines were applied to structure this review. PRISMA-S is an extension of PRISMA 2020, which was originally developed for systematic reviews assessing healthcare interventions, but it can also be applied to reviews with different purposes [38]. Moreover, PRISMA-S was designed with a multidisciplinary perspective [39], reflecting the nature of the ‘One Health’ approach driving this review. In fact, this study combines different disciplines among natural sciences like epidemiology and animal health economics with social sciences.

### 2.2. Search Strategy

The search strategy was conducted through an iterative trial-and-error process [40] on CAB Abstracts and it defined a series of keywords that were linked through Boolean operators. Among the keywords, the list of LMICs was developed through the filter published by Cochrane [41]. As the filter was based on the 2019 list of LMICs according to the World Bank [42] for 2020 fiscal year, changes in 2020, 2021, and 2022 LMICs lists were assessed to exclude discrepancies within the filter (the updated World Bank country classification is finalised every year on 1 July [43]). No limits were applied to the search [38] to retrieve as many publications as possible. The keywords are presented in Table 1 and the complete search strategy for all databases is reported in Supplementary Material S1.

### 2.3. Information Sources

Five databases covering the subjects of human and animal health, veterinary medicine, rural development, animal husbandry, biomedical sciences and policy were consulted to assess the published literature on the topic: CAB Abstracts, EMBASE, Web of Science Core Collection, BIOSIS Citation Index and Scopus. Databases from the same platform were searched one at a time. A librarian from the University of Edinburgh peer-reviewed the search strategy and adapted it to the different platforms as recommended by Spry and Mierzwinski-Urban [44]. Some syntax adjustments like the replacement of question mark with truncation* were applied to the Cochrane filter [41] to better adapt it to the different platforms. Boolean operators were also adapted according to each database requirement. The amended versions of the filter that were applied to the different platforms are reported in Supplementary Material S1. The last search was conducted on 2 September 2023. Backward citation searching of eligible publications was performed. The table of contents of whole proceedings and Special Issues that resulted from the database search and whose subject was rated as relevant were also screened.

### 2.4. Eligibility Criteria for Publications

Publications were included in the review if they had the socioeconomic impact of diseases as the main focus or if they discussed this topic as a collateral subject when the main purpose of the article was different. Papers were considered eligible if the targeted population was represented by working equids like horses, donkeys and mules. Articles that appraised the socioeconomic impact of diseases in various species or in draught animals including working equids were accepted as long as the information could be retrieved individually for the category ‘working equids’. All types of diseases such as non-infectious and infectious diseases, including zoonoses, were considered for this review. Papers were included in the review if the study was set in LMICs according to the current World Bank classification [45]. Full-text peer reviewed publications were rated as eligible for this review. Full-texts from non-peer reviewed literature such as proceedings and organisation reports written and conducted in the form of a research study were also considered eligible for the review based on the recommendations from Benzies et al. [46] and Hartling et al. [47] on the relevance of grey literature in adding evidence to scientific research. Only papers representing original research were included in the review. If appraising the socioeconomic impact of diseases was not the main purpose of the article, reflections made on the socioeconomic impact of diseases had to be based on the study results for the paper to be selected. Articles defining models based on secondary data were considered original research, while reviews were ineligible. Papers written in English, French, Italian, Spanish, and Portuguese were included in this review. Papers targeting working equids belonging to the army or police were not considered for this study as its focus was to understand the impact of diseases on the livelihoods of working equid owners. Articles that mentioned the socioeconomic impact of diseases in working equids as a justification to undertake the study but did not develop the concept further were excluded from the review.

### 2.5. Screening of Sources of Evidence, Data Extraction and Data Items

The results obtained from the database searches were stored on EndNote, deduplicated and selected according to the inclusion criteria. The selection of the results retrieved from the search was carried out in three stages by the first author. Titles were examined at first, followed by the abstract and full texts. Publications that fulfilled all the inclusion criteria were downloaded from online sources or obtained through the digitalisation and inter-library loan services of the University of Edinburgh. The following data were extracted from eligible publications through a data collection form in Microsoft Word (2311) [48], then entered in Microsoft Excel (2311) [49] and synthetised: publication details (peer reviewed/non-peer-reviewed paper, publication source, publication subject, year of publication, language, open access/non-open access), study details (main focus, species, infectious/non-infectious disease, zoonotic/non-zoonotic disease, pathogen type, method to appraise the socioeconomic impact of the disease, type of impact appraised, data collection method, gender balance within study participants, main findings including economic and social effects of the disease and recommendations, presence of any considerations regarding ‘One Health’), study setting, country income status according to the World Bank [45], authors’ affiliation and location and type of funding source. Since descriptions of methods to appraise the socioeconomic impact of diseases were not consistent among studies, information on methods was extrapolated based on the explanations provided within eligible studies. Methods were reclassified according to the definitions of Thrusfield and Christley for cross-sectional studies, cross-sectional surveys, and case studies [50], Scott-Samuel for impact assessment in healthcare [51], Alders et al. for participatory methods [52], and Dijkhuizen, Huirne and Jalvingh for economic models [53]. Among the data collection tools, informal communications were described as a short exchange of communication between researcher and animal caretaker by adapting the definition of Burm et al. [54]. The reclassification enabled the authors to appraise which were the methods most frequently selected by researchers.

## 3. Results

### 3.1. Identification of Eligible Publications

**Figure 1:**
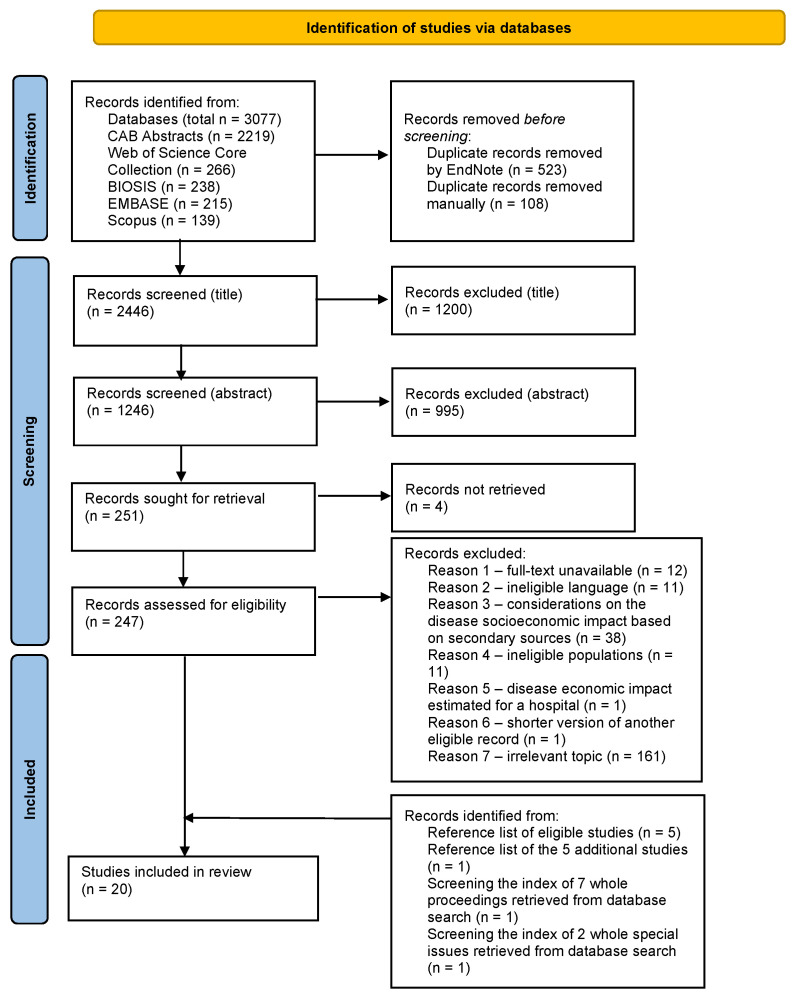
PRISMA 2020 flow diagram for systematic reviews adapted from Page et al. [38].

A search of five databases for academic research identified 3077 publications that, after automated deduplication by EndNote, were reduced to 2554. Following a manual deduplication, the citations deemed suitable for title screening were 2446, from which 1200 publications were excluded because of an irrelevant topic. After the analysis of the abstracts, 995 publications were excluded for an irrelevant topic (993) or because they referred to whole books (2). In total, 251 publications were identified for detailed full-text evaluation. 235 publications were excluded because they did not meet the inclusion criteria, while the full-text of four publications could not be found. At the end of the full-text analysis, twelve eligible publications were identified. Additionally, the index of seven whole proceedings and two Special Issues that resulted from the database search and whose subject was rated as relevant for the purpose of this review, were screened. One eligible publication was obtained from the index of proceedings and one from the Special Issues. Afterwards, five additional relevant articles were obtained from backward citation searching. The reference lists of these five articles were also reviewed and one further eligible publication was identified. Overall, a total of twenty publications were included in the review (the complete reference list is reported in Supplementary Material S2). The phases of the identification of eligible publications are presented in Figure 1:

### 3.2. Features of the Sources of Evidence

An outline of the main features of the eligible publications including publication details, study details, study setting, authors and funding is presented in Table 2:

### 3.3. Synthesis of Results

**Figure 2:**
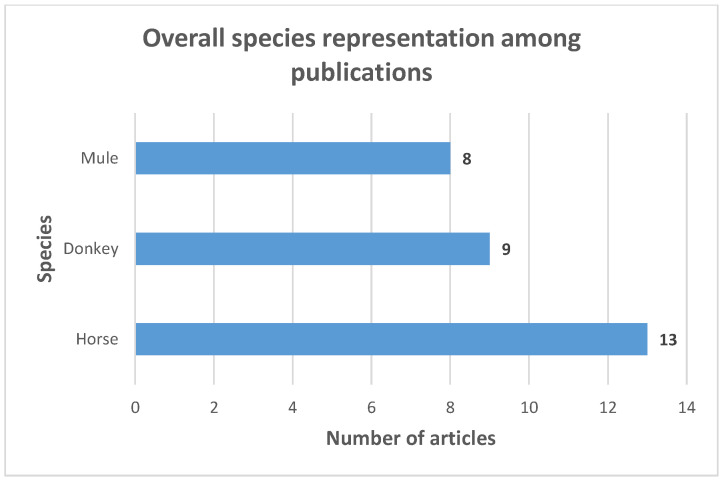
Overall representation of species within eligible publications including articles targeting multiple species.

The majority of publications included in the review (80%) were peer-reviewed. Articles were mostly published from 2010 onwards (85%), they were all written in English and 16 of them were open access. The primary focus of nine articles was different from the socioeconomic impact of diseases, that was covered as a collateral subject. Six articles had the socioeconomic or economic impact of diseases as their main focus. Among them, two papers assessed the economic impact of vaccines and of disease treatment options where the disease economic impact was an essential baseline information for undertaking both studies. Lastly, five articles were focused both on the socioeconomic or economic impact of diseases and on an additional subject with the same level of depth. The majority of publications (14/20) targeted only one species of working equids, while 4/20 publications covered the three equid species. Overall, horses were the most represented species (Figure 2).

**Figure 3:**
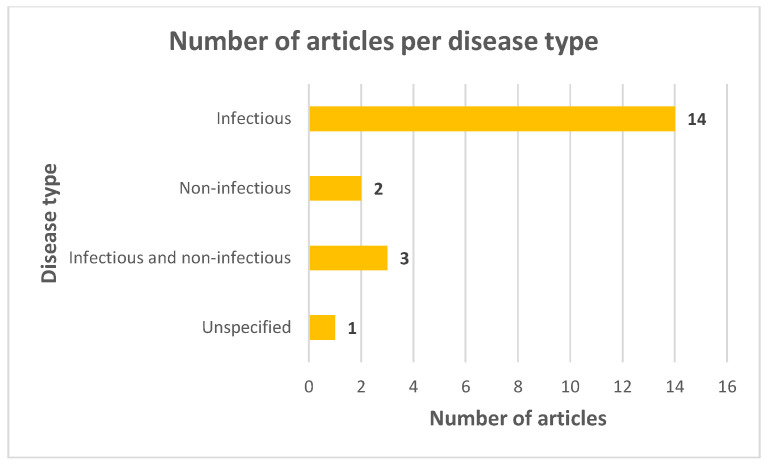
Number of eligible articles per disease type.

Twelve publications investigated an individual disease, three covered various diseases of different types and four studied multifactorial conditions (foot problems, lameness) or diseases determined by the same group of pathogens (endoparasites, fungal diseases). One article referred to disease in general. Overall, infectious diseases were the subject of most of the publications (Figure 3).

**Figure 4:**
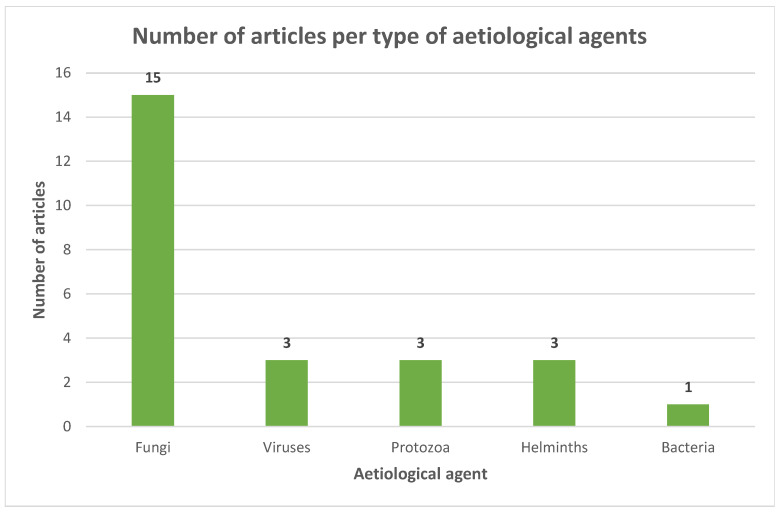
Number of articles per type of aetiological agent among publications that studied only infectious diseases.

Viral, protozoal and fungal diseases were studied individually by twelve articles, with epizootic lymphangitis as the most represented one. None of these diseases were zoonotic. One publication investigated various fungal diseases: among them, two agents were zoonotic, but their zoonotic potential was not discussed [59]. One article studied helminth, protozoal and bacterial agents [15], however, their taxonomic specification was lacking or very general (Table 3, Table 4, Table 5, Table 6 and Table 7). Overall, fungal diseases were the most frequently studied (Figure 4).

The three publications that covered both infectious and non-infectious diseases were not primarily focused on diseases nor on their socioeconomic impact [12,55,63]. In fact, diseases were only mentioned by owners as the cause of their reduced income. This information was incorporated within the broader study aim to assess the contributions of working equids to people’s livelihood or to evaluate the impact of accessing veterinary services on livelihoods. In addition to infectious diseases (Table 8) and non-infectious diseases like foot problems, wounds, colic, eye diseases, dental disorders and sarcoid, respiratory disorders and dermatological problems were mentioned without specifying their infectious or non-infectious nature.

Overall, ten publications evaluated the socioeconomic impact of diseases and ten publications evaluated their economic impact. Among the methods to assess the socioeconomic or economic impact of diseases, cross-sectional and participatory methods were the most frequently adopted, while one publication applied an unstructured method. The economic and social impacts of individual diseases or multifactorial conditions or diseases caused by the same group of pathogens that were covered by the eligible publications are summarised in Table 9.

**Figure 5:**
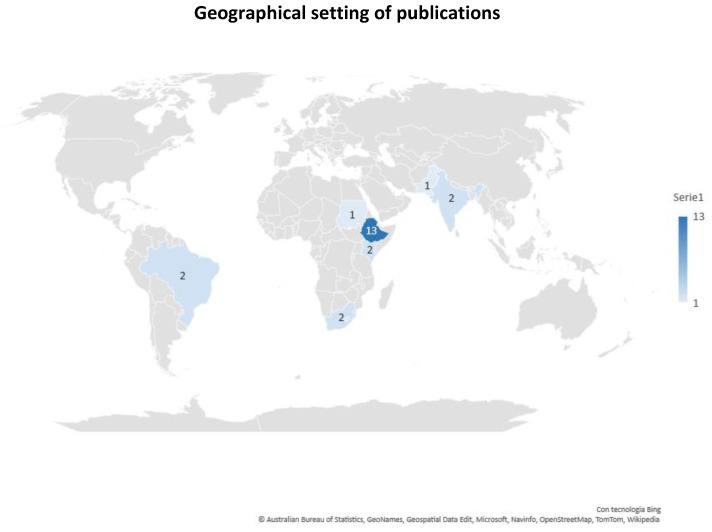
Geographical setting of the publications considered for the review, including the multiple-country study. The blue intensity increases with the number of articles set in each country.

Only six articles reflected on the female-gender perspective, intended as the women’s viewpoint on issues related to working equids. Nevertheless, this aspect was presented through different levels of depth. Only one publication focused on the female-gender perspective in relation to the role of working equids [72], while in another article the female-gender viewpoint was broadly discussed [55]. In one publication, researchers reported that the female-gender perspective was not relevant to the study setting, but it should be considered in other contexts [58]. In one paper, a limited number of females were included among the study participants and there was no reflection on this aspect [65]. According to the authors of another article, women were too reserved to interact with male interviewers, for this reason they were not involved in the study [63]. In one article, researchers recognised the bias of having interviewed only two females and suggested a more balanced gender inclusion among study participants in future research [69]. In regard to the geographical settings of the studies, 19 publications were set in a single country: the most represented country was Ethiopia, where 12 studies were undertaken. The majority of studies (70%) were set in East Africa and, in general, in the African continent (60%). One publication covered multiple continents and countries such as India and Pakistan (Asia) and Ethiopia and Kenya (Africa) (Figure 5). Most of the studies were set in low-income contexts, all corresponding to African countries such as Sudan and Ethiopia.

In 15 publications, both first and last authors were based in the same country where the study was undertaken. Most of these authors belonged to academic institutions (57%), followed by governmental institutions (29%) and working equid charities (14%). In five publications, both authors were based in a foreign western country with a high-income status such as United Kingdom (UK) (4) and United States (US) (1). Regarding the funding sources, the government and working equid charities sponsored most of the publications (25% and 20%, respectively), while for 30% the funding origin was unspecified. One study did not require any financial support. Among the seven publications where funds were sourced from a foreign country, in five cases the support originated from UK-based charities. One publication was funded by the British Department for International Development, while another one was sponsored by multiple international organisations and a UK-based research centre (Pirbright Institute). Overall, the UK contributed to all the externally funded publications and fully funded six publications. Among the six publications whose funds originated from the same country as the study setting, most of the funds came from the government (4), followed by an academic institution (1) and a national equine veterinary association (1). In Ethiopia, only one publication was locally funded, while five publications had an unspecified source of funds and were issued by academic institutions. All studies set in Brazil were locally funded by the government. Most of the studies whose funds were sourced locally were set in upper-middle income countries. Although few studies were conducted by multidisciplinary teams, none of the publications applied the ‘One Health’ approach or recommended it for future studies or interventions. Nevertheless, in one article, Duguma et al. recognised the potential advantages from engaging a multidisciplinary team in future research combining epidemiology, social science, and economics expertise [58]. In regard to the three domains of ‘One Health’, human, animal, and environmental health, all publications focused on animal health, while no reference was made to the health of the ecosystem. Two publications highlighted the link between the decreased income-generating capacity of sick animals and the potential effects on human health because of the reduced ability of households to afford health services and nutritious food [63,72]. Lastly, two papers reported mental health consequences on owners of animals affected by epizootic lymphangitis [57] and African horse sickness [60].

## 4. Discussion

While the socioeconomic contribution of working equids to livelihoods has been widely demonstrated [1,73,74], this review shows that the socioeconomic impact of diseases of working equids in LMICs is a topic that still lacks attention and research is restricted to few diseases and geographical settings. With Ethiopia as the most represented country among the results of the review, the study setting distribution partly reflects the population trends of working equids worldwide, where Ethiopia has the largest working equid population [4]. The heavy burden of infectious diseases on working equids [75] explains why the articles focus mainly on infectious diseases. Whilst limited in number, the publications included in this review represent a starting point for future research and for informing policy under a ‘One Health’ perspective.

### 4.1. Considerations on Species of Working Equids

Horses were the most represented species among the publications included in this review. This is probably to attribute to their higher economic value compared to mules and donkeys. However, in some contexts like Ethiopia, mules can have high market prices due to their working power and the high socioeconomic status attributed to mule ownership [55]. The prioritisation of horses may have occurred also because donkeys are usually given less attention in terms of veterinary care [76] since they are considered more resistant to diseases [77]. These aspects have probably guided the choices of diseases to study made by authors. The lower research interest for donkeys and mules is supported by the fact that they are targeted primarily by articles covering multiple species rather than individual species. Overall, mules may be less represented since their population figures are lower compared to other species among the country settings, except for Brazil and Ethiopia, that have high mule populations [4]. In fact, three studies set in Ethiopia targeted mules individually [56,57,58], reflecting their high socioeconomic value in addition to their population size. In the Ethiopian context there was a discrepancy between equid population figures and interest in donkey-related research since the donkey population is the highest compared to other working equids [4], while donkeys were targeted individually only by two publications [63,71]. In Ethiopia, donkeys are known to be preferred by women because they are easier to handle and they provide the most significant contribution in reducing their work burden [55]. In this case, the neglect of donkeys in research is in line with the very limited consideration of females’ perspective by the eligible publications. What affects women tends to receive less attention, although owning an equid is more beneficial for women-headed households than for male-headed households [55].

### 4.2. Considerations on Diseases

#### 4.2.1. Infectious Diseases

Although working equids are affected by a variety of infectious and non-infectious diseases [12,78], the publications included in this review were primarily orientated towards infectious diseases (Figure 3). This reflects the high morbidity and mortality of infectious diseases and their effects on the animal working capacity. Among the infectious diseases covered individually by the eligible publications, only equine infectious anaemia and African horse sickness are WOAH-listed diseases. However, official disease status can only be reported for African horse sickness [79].

Epizootic lymphangitis was rated by equid owners as the most critical and fatal disease affecting their equids, causing poverty and unemployment [58,61]. This is confirmed by the high prevalence of epizootic lymphangitis recorded by epidemiological studies [80,81]. Contrastingly, owners’ knowledge on the disease was lacking [69]. All authors of eligible publications agreed that economic losses were linked to animal death and to the decreased work productivity of affected animals due to reduced worked time and load capacity. Bekele et al. recorded that the average income generated by a sick mule was slightly higher than half of the income produced by a healthy mule [57]. Similar findings were reported by Nigatu and Abebaw [66] and by Mitku, Assefa, and Abrhaley [64] for horses. Moreover, costly treatments with poor outcomes resulted in animal death, abandonment and disease relapse that induced owners to replace their animals [57,66]. Because of skin lesions, animals were unable to be sold [59] or clients refused to hire them [64]. The disease also had a social impact because owners were stigmatised due to the infectious nature of the disease and the poor appearance and bad smell of the animals [57]. The owner’s loss of motivation and mental health consequences can also be considered as social impacts of the disease. Because in this context it is difficult to change profession or to obtain a loan to replace animals, these conditions increase the precariousness of the livelihood of equid owners and initiate a poverty cycle that is difficult to interrupt [57,69]. Other social implications were the negative impact on the image of cities because of sick stray equids causing traffic accidents and difficulties for the waste collectors to remove animal carcasses [58]. Among the recommendations to address epizootic lymphangitis, several authors indicated participatory interventions for sustainable disease control and prevention [57,66]. For example, the intervention implemented by Duguma et al. resulted in a considerable decrease in disease prevalence [58]. Since all publications on epizootic lymphangitis were concentrated in Ethiopia, more research is needed to understand its epidemiology and socioeconomic impact in other contexts where the disease is reported like Iraq [82], Sudan [83], Senegal and South Africa [84]. However, the disease may be highly underreported: while in Ethiopia it is obviously present [80,81], no reports are displayed by the World Animal Health Information System and this may also apply to other contexts [84]. For this reason, favourable climatic conditions of humidity, rainfall and temperature [81] should also be considered when targeting locations for future studies. Research on how climate change may affect the disease epidemiology should be undertaken.

Although African horse sickness is endemic in various LMICs in Sub-Saharan Africa [85,86] and it causes high mortality rates among working equids in Ethiopia [87] and Senegal [88], the disease was targeted only by two publications set in South Africa [60,68]. Direct losses related to animal deaths were estimated by Redmond, Jones, and Rushton as 500,000 USD yearly [68]. Grewar et al. highlighted indirect losses of equids’ breeding potential and of previous investments in horse care as well as mental health consequences for their owners [60]. To inform further studies on the socioeconomic impact of African horse sickness, research on the epidemiological situation in areas with large working equid populations is recommended [68]. In fact, it should be considered that in many of these countries, especially in Sub-Saharan Africa, there is no official status for African horse sickness [79], while several outbreaks have been reported since 2017 in Chad, Senegal, Nigeria, Ethiopia, South Africa, Namibia, Zimbabwe, and Malawi [84].

Equine trypanosomiasis (surra) is widely distributed across Asia, Latin America, and Africa [89] and it determines severe consequences on working equid health including emaciation, anaemia, neurological signs and death [90,91]. Nevertheless, very limited research is available on its socioeconomic effects. Kumar et al. created a multispecies model for India where equid related losses were estimated as 2.20 million USD yearly, including 933,254 USD lost in traction power [62]. Seidl, Moraes, and Silva developed a model targeting equids working within the Brazilian beef industry. The authors estimated the economic impact of trypanosomiasis based on different treatment and prevention strategies, where whole-year treatment was found as the most convenient option [70]. Without treatment, the yearly losses calculated on the entire Pantanal horse population were estimated as 2,400,000 USD, corresponding to the death of 6462 horses. This could be translated into over 2000 USD yearly losses per ranch, with an average of six horses that died [70]. An ineligible study conducted in a similar context in Venezuela confirmed the profitability of trypanosomiasis treatment and estimated yearly financial losses of 7,486,000 USD without treatment [92]. Severe economic effects of surra emerged also from a publication set in a region of Indonesia which was not retrieved by the database search. The authors estimated the costs of an outbreak for the working equid sector as 661,000 USD including treatment, animal culling and production losses [93]. Studies on the socioeconomic impact of surra should be extended to more LMICs where the disease vectors *Tabanus* spp. (horse flies) and *Stomoxys* spp. (stable flies) are present. However, epidemiological data is lacking and surra is often underreported, causing an underestimation of the disease burden [84,89].

In regard to quine infectious anaemia, the increase in the prevalence of the disease that was recorded among working equids of the Brazilian Pantanal Region, the consequent travel ban, and stigmatisation of affected animals (estimated as 13,000) determined a decrement of equid prices. These findings, associated with the reduced performance of sick equids, can be translated into severe economic consequences for their owners [67]. Because of the higher exposure to the vector and lack of hygiene, working equids are at higher risk of contracting equine infectious anaemia compared to other equids. Hygiene promotion to prevent harness sharing and disease testing to isolate seropositive animals are recommended to control the disease and limit its socioeconomic effects [67,94].

While working with donkeys is highly profitable in Sudan, poor attention is given to disease prevention and the presence of gastrointestinal parasites has been associated with a reduced daily income [15]. Similarly, Kenyan owners reported that income was negatively affected by gastrointestinal parasites because of decreased animal efficiency and high treatment costs [12]. Education programmes highlighting the positive impact of deworming on earnings could promote owners’ behavioural changes [15].

#### 4.2.2. Non-Infectious Diseases

Lameness and foot diseases like overgrown hooves and hoof abscesses are very common in working equids [2,95] due to overloading, overworking, poor hoof care, working at premature age and inappropriate harness and cart. All these risk factors could be prevented by improving owners’ knowledge on animal care and sustainable harness [20,56,71]. In Ethiopia, the average annual loss due to foot disorders in donkeys was estimated as 123.45 USD, based on non-worked days and treatment costs. The owner that reported the longest recovery period for his animal (60 days) lost 387 USD in one year. These losses are very severe considering that the average yearly income generated by a donkey was recorded as 1488 USD [71] and that the Ethiopian average yearly income is 890 USD [96]. Similarly, 5% of mule owners interviewed by Ali et al. reported that their animals had no economic value due to lameness [56]. In another article, Kenyan owners associated a reduced income to foot disorders [12]. Investments in owners’ education, extension services and advocacy among governments to enforce animal welfare regulations are the way forward to prevent lameness and hoof disorders [97]. Participatory programmes have been successful in preventing lameness by delivering knowledge and influencing management changes among working equid owners [20,98].

#### 4.2.3. Other Diseases

Some publications covering multiple diseases provided general information about the socioeconomic impact of infectious and non-infectious diseases that could represent potential subjects for future research. For example, colic and wounds, that are highly prevalent in working equids [56,99], were classified by owners as diseases with fatal outcome that decreased the family revenues. Similarly, respiratory disorders reduced the daily income because of poor animal efficiency [12,55]. Skin diseases like mange, besides having high treatment costs, affected the animals’ appearance, reducing their market price and their income-generating capacity because clients were unwilling to hire these animals. This issue had also social implications resulting in owners’ marginalization [12]. Tetanus was mentioned by owners as a very frequent problem, causing high financial losses due to expensive treatment and animal fatalities [12]. Tetanus occurs very often in working equids because vaccines are unavailable in LMICs [18]. Moreover, it is associated with tethering and tack wounds, that are commonly observed in working equids [100,101]. The lack of access to veterinary services aggravates the situation because without treatment the disease is fatal [102]. For these reasons, research on the socioeconomic impact of tetanus should be urgently undertaken to promote tetanus vaccination in working equids. Among the diseases reported by owners in eligible publications covering multiple diseases, dental disorders may have severe economic consequences due to their secondary effects such as weight loss and colic [99,103]. Since dental disorders are preventable diseases, evidence of their socioeconomic impact could facilitate investments in the education of owners and veterinary personnel and in the distribution of dental equipment to veterinary facilities [104].

#### 4.2.4. Zoonotic Diseases

Although none of the infectious diseases whose socioeconomic impact was analysed in depth was zoonotic, some zoonoses were mentioned within publications covering multiple diseases. Working equid owners are highly exposed to zoonoses because of the close contact with their equids and other livestock with whom they often share their living space and water sources, especially in slums and urban areas [105]. Due to their implications on animal and human health, research on the socioeconomic impact of zoonoses like leptospirosis and glanders is highly recommended. In urban areas, working equids are particularly at risk of contracting leptospirosis because of the poor hygiene of stables and working environments where they have contacts with rodents and other domestic animals [105]. Since working equids can be a source of infection for their owners, leptospirosis may determine severe socioeconomic effects among working equid communities [21,106]. Moreover, poor hygienic conditions are evidence of owners’ lack of knowledge on the zoonotic potential of the disease [21]. Economic effects of leptospirosis in equids could be linked to the severity of the acute form and to the reduced animal efficiency in the most common subclinical form [107]. Glanders affects working equid populations [77] with occasional outbreaks in Asia, the Middle East, and South America and it is a WOAH-listed disease [108]. Glanders has a potentially high socioeconomic impact because it reduces equid performance and requires the culling of positive animals as a control measure. Additionally, it is an occupational illness, that, if left untreated, is fatal [109,110]. In Pakistan, the low compensation prevents owners from testing and culling positive animals [111]. For this reason, research on the socioeconomic impact of glanders in working equids should be undertaken to sensitise governments to provide appropriate compensation [109]. This could enhance effective disease control and reduce the economic losses [112].

### 4.3. Methods

The participatory method was one of the most preferred approaches among eligible publications. The participatory method has been broadly applied in LMICs to promote community contributions in identifying issues related to human [113] and animal health [114,115], including the health of working equids [116]. Within eligible articles, the participatory approach facilitated the understanding of the social context and of owners’ knowledge and perception of diseases and of their socioeconomic impact [69]. For these reasons, programmes informed by participatory research are more sustainable, like the one implemented for the prevention and control of epizootic lymphangitis by Duguma et al. [58]. Since participatory approaches are tailored to particular contexts [78], they cannot be identically replicated but need adaptation to the targeted area, making comparison between studies more difficult. For this purpose, participatory research can be integrated with quantitative research such as cross-sectional studies [78], as applied by some eligible publications. A mixed approach can ensure that policy and interventions are comprehensively informed by objective quantifications, by the prioritisation of needs made by the communities and by their perception of animal health problems [116].

Although the scope of participatory research is highlighting the view of the community on a particular issue [114], plurality within groups involved in participatory research, for example in terms of female-gender representation, is often lacking [117]. This is confirmed by the absence of women’s perspectives in nearly all the publications and by the scarce number of females interviewed within most of those publications that attempted to include women within participatory groups. The perspective of age groups was also not considered among publications. In one article, researchers selected only elderly people for the interviews, thinking that they had a broader experience on the subject [63]. The inclusion of people with disabilities, chronic illnesses, and people from ethnic minorities was not applied by any of the publications. This may result in a biased community voice. Some authors could argue that engaging women may not be appropriate or feasible in certain settings [63] or that including people with illnesses may expose them to stigmatisation and isolation from the community [118]. Some challenges can be mitigated by involving female researchers, that would not only facilitate access to women within communities but would also foster equality within research teams [119]. Nevertheless, it should be highlighted that in some contexts, regardless of the availability of female interviewers, women may not be able to participate to interviews due to cultural and religious reasons and they may need to seek for the approval of a male family member to attend the interview [120]. A thorough stakeholder mapping can help identifying groups or associations of people with illnesses and disabilities that may facilitate approaching individuals in a confidential manner.

### 4.4. Socioeconomic Effects of Working Equid Diseases on the Livelihoods and Health of Women

Health problems and the death of working equids have severe effects on women. The most detrimental effects are on households headed by women who are supported by working equids for income-generating activities or in areas where women are fully in charge of child and animal care, water and firewood collection or food purchase [55]. Women heads of households without a source of income may resort to negative coping strategies like prostitution [121,122] to cover their basic needs. The lack of access to a balanced diet and the heavy work burden may affect the health of women, including their mental health [72]. Children and young people belonging to these households without access to education and living in poverty may be more in danger of radicalisation [123], recruitment as child soldiers [124] and illegal migration [125]. Due to the loss of their equid, women may take a longer time to complete household tasks like fetching water. In this way their time for childcare is reduced with negative consequences on the health of children. Moreover, women lose the opportunity to dedicate to income-generating activities as well as to social activities [72,126]. This makes women-headed households more vulnerable in addition to increasing gender gaps [55].

### 4.5. Study Setting

Overall, the distribution of study settings reflects the global equid population figures, where Ethiopia is the first country for the number of equids and eligible publications. This could be ascribed to the crucial contribution of working equids to livelihoods and to the Ethiopian economy [5,127]. Research promotion by the Ethiopian government [128] and the large number of veterinary schools [129,130] may have impacted positively the research output. Moreover, the presence of working equid charities such as Brooke [131], The Society for the Protection of Animals Abroad (SPANA) [132] and The Donkey Sanctuary [133] may have influenced research orientation since eight Ethiopian publications were linked to charities for financial support, authors’ affiliation or collaboration with charity projects. Nevertheless, some may argue that the charity presence does not affect the research output. In fact, while the same charities are present in India, only two among the eligible articles were set in India and only one was published by a charity [72]. While working equids represent an important livelihood asset in India [9], the scarce number of publications may reflect the limited recognition of the socioeconomic role of working equids compared to other working animals like oxen, that are often targeted within articles on disease socioeconomic impact [134,135]. The English keyword search may have missed publications in French, for example from French-speaking African countries such as Chad, that is the sixth country with the largest equid population worldwide [4].

### 4.6. Authors

Most of the authors of eligible papers were based in the countries where the studies were undertaken. This demonstrates that this area of research is directed towards a decolonisation process. Nevertheless, this consideration applies to a small sample size and current research inequalities should not be underestimated. LMICs should not just be seen as a fertile source of data for western researchers [136]. Where expertise is present, it must be recognised and local authorship promoted [137,138] so that locally produced research can potentially influence policy [139]. Where capacity needs to be built, researchers from LMICs should be integrated into research groups on an equal opportunity basis [138], as it is applied for example within the Transboundary Research Partnerships approach of the Swiss Academy of Sciences (SAS) that promotes equal and sustainable research collaborations with LMICs [140]. To facilitate research output from LMICs and ensure research equality, it is essential that donors and academic institutions in high-income countries provide financial support to researchers in LMICs and revise their research collaboration policies [138].

### 4.7. Funding

The considerable number of publications where the funding source remained unknown may bias any consideration made on funding sources. However, it can be extrapolated that while some governments invest in research targeting working equids, support from charities is essential. The interest of the Brazilian government in funding working equid-related research may be linked to the crucial role of equids in the cattle industry [141] that widely contributes to the national economy [142]. The WOAH provided a financial contribution only to the study by Redmond, Jones, and Rushton where the discussion on the benefits of a vaccine against African horse sickness focused on the international equine industry [68]. This explains also the interest of the International Federation of Horseracing Authorities (IFHA) and of the Federation for Equestrians Sports (FEI) in funding the study. These findings may suggest that working equid research is rarely considered for resource allocation by international organisations and governments unless there are some implications for large-scale and international sectors or for the national economy. The UK remains the main financial contributor to this research field probably because all the charities are UK-based and because of the role of equines in British history, economy and traditions [143,144]. These aspects may have promoted the acknowledgement by British donors of the important role of working equids in LMICs.

### 4.8. Socioeconomic Effects of Working Equid Diseases: The ‘One Health’ Perspective

Although none of the articles applied the ‘One Health’ approach to evaluate the socioeconomic impact of diseases, the findings of this review demonstrate that the health of working equids is an issue of ‘One Health’. Working equid diseases cause detrimental effects on animal welfare, reduce the animals’ working efficiency and, consequently, their income-generating capacity. This causes indirect implications on human health and wellbeing since the household economy is disrupted and basic needs like nutritious food and healthcare cannot be covered. The compromised health of working equids affects all the activities depending on them, from ploughing to carrying goods to the market or driving children to school, weakening the household resilience [1,9]. The family living conditions are negatively impacted, determining mental health consequences on family members or on the household head that is no longer able to support the family [12,57]. Moreover, while no zoonotic diseases were analysed in depth within eligible publications, zoonoses of working equids can directly affect human health [21,145]. Limited availability of water because of illness of working equids can compromise the hygiene of households and promote the spread of faecal-oral transmitted diseases [146]. As a coping strategy, households may use unsafe water with a potential spread of water-borne diseases such as diarrhoea, which represents the second cause of mortality in children under five years old [146].

In addition to the health and welfare of working equids, the health of other livestock who depend on them to receive water, feed and veterinary care is impacted [1]. This can be translated into food insecurity, because of the loss of economic assets and animal products that could directly feed the family. The health of the ecosystem is compromised where working equids engaged in waste management are incapable to work and garbage accumulates in the environment [14]. Undisposed waste can also lead to consequences on human health due to the release of toxic gasses and to the proliferation of disease vectors such as flies and mosquitoes. Undisposed waste can create a blockage of water drains that results in stagnant water and in the potential spread of water-borne diseases such as cholera and vector-borne diseases such as malaria, due to increased presence of mosquitoes [147,148]. Moreover, in case of equids unavailability due to illness, people may resort to motorised vehicles, with a consequent increase in carbon emissions and damage to the health of the environment [149].

Given these considerations, applying the ‘One Health’ approach to research would better frame the multiple implications that working equid diseases have on human, animal and environmental health. This could facilitate the acknowledgement of the importance of addressing working equid health problems among policymakers within governments and international organisations in LMICs [9]. However, Spencer et al. could argue that these transdisciplinary collaborations still lack guidelines, representing an obstacle to the successful implementation of ‘One Health’ research [150]. This challenge can be mitigated by the provision of training to researchers and by reflecting on previous transdisciplinary collaborations. Overcoming these barriers can be an opportunity for African researchers to become the driving force of ‘One Health’ research. According to Kamani et al., multidisciplinary collaborations are more natural for African researchers because they already understand the interdependency among humans, animals and the ecosystem due to the environment they live in [151]. Consequently, ‘One Health’ represents an opportunity for decolonisation and transboundary research collaborations also in the field of working equid health.

### 4.9. Study Limitations

Due to time constraints, some aspects of the review process could not be covered but they can be recommended for a future study or to integrate the current review. Forward citation searching of eligible articles was not applied. This could have identified additional publications [152]. The database search was not conducted with keywords in eligible languages other than English. This could have been particularly meaningful for retrieving publications written in French, especially from West Africa [139]. According to Hartling et al., non-English articles could have influenced the results of the review due to the limited number of eligible publications [47]. Grey literature was not systematically searched in any of the eligible languages and only the publications obtained from the database search or backward citation searching were included. Grey literature could have added evidence to the review [46], especially from non-English speaking contexts and could have also reduced publication bias [37]. National journals that are not indexed by international databases were not screened. Since this is advisable for research targeting LMICs [139], it could have increased the number of eligible articles. However, some non-indexed publications were identified through backward citation searching. While a systematic approach to methods and reporting was applied to enhance transparency and reproducibility of the review [37,38], the risk of bias within eligible publications was not assessed. The articles that met all the inclusion criteria were included in the review regardless of their strength of evidence because, due to the heterogeneity of the study designs, it was not possible to effectively evaluate the quality of evidence.

## 5. Future Directions

Although in limited number, the eligible publications demonstrated the detrimental effects of working equid diseases on animal health, human wellbeing, and livelihoods. Nevertheless, there are still considerable research gaps that need to be urgently addressed. Some recommendations are provided to increase the potential of future research on disease socioeconomic impact to influence policy and to inform programmes aimed at improving the health of working equids. Some proposals for policy and sustainable interventions are also presented.

### 5.1. Recommendations for Future Research

The socioeconomic impact of zoonoses like rabies, glanders, leptospirosis, of WOAH-listed diseases such as equine influenza, of fatal diseases like tetanus and non-infectious diseases like colic and dental disorders that severely affect health and welfare of working equids should be investigated. Research on diseases with demonstrated high socioeconomic impact like epizootic lymphangitis and trypanosomiasis should be extended to more contexts where there are optimal climatic conditions, vectors and large working equid populations. While eligible publications privileged horses, more studies targeting donkeys should be undertaken, especially in contexts where they are numerous and in relation to their contribution in reducing the work burden on women. Since working equids are severely neglected in countries affected by insecurity and conflict, research should be conducted in these contexts to improve policymaking. Involvement of extension agents who have access to outreach areas could facilitate data collection besides strengthening the disease surveillance system.

Research should be guided by the ‘One Health’ transdisciplinary approach to ensure that the multifaceted socioeconomic implications of working equid diseases on human, animal and environmental health and welfare are appreciated. Institutionalisation of ‘One Health’ within working equid charities could be a starting point to promote research on working equids under a ‘One Health’ perspective and it could also have a positive influence on policymaking. To provide detailed information to policymakers, mixed methods combining quantitative data and qualitative information gained through participatory methods, are recommended. Capturing the community viewpoint through the participatory approach can ensure that priority diseases and their socioeconomic effects are not overlooked. The inclusion of the perspective of disadvantaged categories like women, people with disabilities and illnesses is essential to providing a comprehensive community voice. This is in line with the need to incorporate women, minorities and indigenous people’s perspectives within ‘One Health’ research, since they can lead to more sustainable ways to address complex issues [153]. In this regard, it is also essential that research teams are diverse. The presence of female researchers can facilitate communication with women within participatory groups. Due to the multiple implications of working equid diseases, it is recommended to publish research on this subject in ‘One Health’ and multidisciplinary journals. This could enhance dissemination of information and increase the potential of research to influence policy. Open access journals should be selected to facilitate access to publications from researchers and policymakers from LMICs.

### 5.2. Recommendations for Policy and Interventions

This review shows that, by protecting the health of working equids, their support to livelihoods could be maximised in terms of efficiency and length of service. This could save treatment and animal replacement costs and it could interrupt poverty cycles with consequent benefits on animal welfare, human health, and wellbeing as well as on national economies. Based upon this, it is recommended that government and international organisations operating in LMICs design policies aimed at:

- Improving the surveillance of working equid diseases with provision of regular reports to WOAH.
- Promoting funding allocation for prevention and control of working equid diseases, especially in conflict affected areas where charities are not operative. This could be achieved through immunisation campaigns, distribution of drugs and equipment to government clinics and extension agents, education of owners, veterinary staff and community animal health workers on working equid care, biosecurity and sustainable harness, training and enforcement of drug store workers. Provision of free veterinary services where communities are particularly vulnerable should be considered.
- Inclusion of health and welfare of working equids within curricula for community animal health workers, veterinary and agricultural degrees.
- Ensuring that women, people with disabilities, chronic illnesses, and minorities benefit from programmes targeting working equids.

For implementing interventions targeting working equids, participatory projects are recommended. In fact, participatory projects have produced positive changes since they empowered communities and stakeholders to recognise equid health problems and to find sustainable solutions to address them [58]. The integration of the ‘One Health’ approach could ensure that projects are designed to improve animal, human, and environmental health. While emphasising the need for government and international organisations to take responsibilities for working equid health, it should not be underestimated that they may lack technical expertise in a field that is quite specialist. When needed, working equid charities should be engaged in policymaking. Moreover, charities should have a leading role in building the capacity of government and organisations’ veterinary staff on how to conduct sustainable interventions aimed at improving working equid health and welfare.

## 6. Conclusions

This review has demonstrated that diseases of working equids have devastating effects on animal welfare, human wellbeing, and livelihoods in LMICs because they reduce the capacity of households, especially women-headed households, to cover their basic needs and they weaken their resilience. Nevertheless, working equids are often excluded from animal health policies and interventions by governments and international organisations in LMICs, that ignore their crucial contribution in reducing poverty. To create awareness among policymakers about the multiple benefits of protecting the health of working equids, more research is needed on the socioeconomic impact of their diseases. As shown by this review, this subject is still under-researched. While most of the publications focused on the socioeconomic impact of epizootic lymphangitis in Ethiopia, there are several diseases that, because of their high morbidity and mortality like equine trypanosomiasis or their zoonotic potential such as glanders and leptospirosis, should be investigated in contexts with high populations of working equids. Due to the complex implications of working equid diseases that emerged from this review, the ‘One Health’ approach is particularly suitable to study this subject area, where a transdisciplinary perspective could provide more clarity on the link between working equid health and human wellbeing, facilitating the translation of research into policy. The consideration of working equid health needs within animal health policy is essential to securing funding allocation for interventions aimed at protecting the health of working equids and, consequently, the health and livelihoods of the communities depending on them. ‘One Health’ is an opportunity to make working equids more visible and to promote transboundary and equal research collaborations lead by researchers from LMICs. Lastly, integrating the perspectives of women and of indigenous people within ‘One Health’ research could promote gender equality and social inclusion of indigenous people while fostering more sustainable solutions to tackling the burden of working equid diseases.

## Figures and Tables

**Table 1:** Search strategy developed for CAB Abstracts. The groups of keywords are combined through the Boolean operator AND. Within each group, keywords are combined through the Boolean operator OR.

Socioeconomic keywords	household *, communit *, income, livelihood *, socioeconomic, econom *, poverty
Disease keywords	disease *, zoono *
Working equids keywords	work *, pack *, plough *, plow *, draft *, draught *, transport, traction *, cart *
	animal *, equid *, equine *, livestock, donkey *, horse *, mule *,
	carthorse *, cart-horse *
Low and middle-income countries keywords	World Bank filter [41]

* both singular and plural forms were considered within the search.

**Table 2:** Main features of the publications included in the review.

Publication Details		Study Details				Study Setting and Authors			
Author (Year)	Publication Source	Main Focus	Disease Name	Species	Method to Appraise the Socioeconomic or Economic Impact of the Disease	Country	Country Income Status	Authors’ Affiliation	Source of Funding
Admassu and Shiferaw (2011) [55]	Organisation report	Socioeconomic role of working equids	Multiple	Horse Donkey Mule	Participatory method; cross-sectional study	Ethiopia	Low income	The Brooke	The Brooke
Ali et al. (2016) [56]	Journal	Disease epidemiology	Lameness	Mule	Cross-sectional survey	Ethiopia	Low income	Cornell University; Washington State University	Unspecified
Angara, Ismail and Ibrahim (2011) [15]	Journal	Socioeconomic role of working equids and disease socioeconomic impact	Helminths and blood parasites	Donkey	Cross-sectional study	Sudan	Low income	Sudan University of Science and Technology; University of Bahri	Ministry of Higher Education and Scientific Research
Bekele et al. (2014) [57]	Proceedings	Disease socioeconomic impact	Epizootic lymphangitis	Mule	Cross-sectional study; participatory method	Ethiopia	Low income	The Donkey Sanctuary	The Donkey Sanctuary
Duguma et al. (2021) [58]	Journal	Description of a community-based intervention	Epizootic lymphangitis	Mule	Participatory method	Ethiopia	Low income	The Donkey Sanctuary	The Donkey Sanctuary
Etana (1999) [59]	Journal	Disease epidemiology	Fungal diseases	Horse	Unstructured method	Ethiopia	Low income	Awassa College of Agriculture	Unspecified
Gichure et al. (2020) [12]	Journal	Socioeconomic role of working equids	Multiple	Donkey	Participatory method	Kenya	Lower-middle income	Chuka University; University of Nairobi	None
Grewar et al. (2013) [60]	Journal	Disease outbreak description	African horse sickness	Horse	Case study	South Africa	Upper-middle income	Department of Veterinary Services of Western Cape Province	South African Equine Veterinary Association
Jagema and Jarso (2016) [61]	Journal	Disease epidemiology and socioeconomic impact	Epizootic lymphangitis	Horse	Cross-sectional study	Ethiopia	Low income	Addis Ababa University	Unspecified
Kumar et al. (2017) [62]	Journal	Disease economic impact	Equine trypanosomiasis	Horse Donkey Mule	Simulation model	India	Lower-middle income	National Research Centre on Equines	Indian Council of Agricultural Research
Martin Curran, Feseha and Smith (2005) [63]	Special Issue	Impact of the access to veterinary services	Multiple	Donkey	Impact assessment	Ethiopia	Low income	Private consultant; The Donkey Sanctuary; University of Aberdeen	Department of International Development, United Kingdom
Mitku, Assefa and Abrhaley (2018) [64]	Journal	Disease epidemiology and socioeconomic impact	Epizootic lymphangitis	Horse	Cross-sectional study	Ethiopia	Low income	University of Gondar	Unspecified
Molla, Fentahun and Jemberu (2021) [65]	Journal	Evaluation of owners’ knowledge and management of a disease and its socioeconomic impact	Epizootic lymphangitis	Horse Mule	Cross-sectional study	Ethiopia	Low income	Mekidela Amba University; University of Gondar	University of Gondar
Nigatu and Abebaw (2010) [66]	Proceedings	Disease socioeconomic impact	Epizootic lymphangitis	Horse	Cross-sectional study	Ethiopia	Low income	University of Addis Ababa	Unspecified
Nogueira et al. (2017) [67]	Journal	Disease epidemiology and evaluation of a disease-control intervention	Equine infectious anaemia	Horse Donkey Mule	Cross-sectional study	Brazil	Upper-middle	Empresa Brasileira de Pesquisa Agropecuária (EMBRAPA), Pantanal	Fundação de Apoio ao Desenvolvimento do Ensino, Ciência e Tecnologia do Estado de Mato Grosso do Sul and Research Center of Pantanal
Redmond, Jones and Rushton (2021) [68]	Journal	Disease economic impact	African horse sickness	Horse	Cost–benefit analysis model	South Africa	Upper-middle income	University of Liverpool	International Federation of Horseracing Authorities (IFHA), International Federation for Equestrian Sports (FEI), World Organisation for Animal Health (WOAH), Pirbright Institute
Scantlebury et al. (2015) [69]	Journal	Disease socioeconomic impact	Epizootic lymphangitis	Horse Donkey	Participatory method	Ethiopia	Low income	University of Liverpool; The Society for the Protection of Animals Abroad (SPANA)	The Horse Trust
Seidl, Moraes and Silva (1998) [70]	Journal	Disease economic impact	Equine trypanosomiasis	Horse	Cost–benefit analysis model	Brazil	Upper-middle	EMBRAPA, Pantanal	Unspecified
Solomon et al. (2019) [71]	Journal	Disease epidemiology and socioeconomic impact	Foot problems	Donkey	Cross-sectional study	Ethiopia	Low income	Hawassa University	Unspecified
Valette and Upjohn (2014) [72]	Proceedings	Socioeconomic role (gender-based) of working equids	Unspecified	Horse Donkey Mule	Participatory method	Ethiopia; India; Kenya; Pakistan	Low income	The Brooke	The Brooke

**Table 3:** Viral diseases studied by publications covering only infectious diseases.

Viral Diseases			
Disease Name	Aetiological Agent	Number of Publications	Zoonotic Potential
African horse sickness	*Orbivirus* genus	2	No
Equine infectious anaemia	*Lentivirus* genus	1	No

**Table 4:** Bacterial diseases studied by publications covering only infectious diseases.

Bacterial Diseases			
Disease Name	Aetiological Agent	Number of Publications	Zoonotic Potential
Anaplasmosis	*Anaplasma* spp.	1	No

**Table 5:** Protozoal diseases studied by publications covering only infectious diseases.

Protozoal Diseases			
Disease Name	Aetiological Agent	Number of Publications	Zoonotic Potential
Equine trypanosomiasis	*Trypanosoma evansi*	2	No
Piroplasmosis	Unspecified	1	No

**Table 6:** Helminthiasis studied by publications covering only infectious diseases.

Helminthiases			
Disease Name	Aetiological Agent	Number of Publications	Zoonotic Potential
Gastrointestinal parasites	Unspecified	1	No
Microfilariasis	*Onchocerca* spp.	1	No

**Table 7:** Fungal diseases studied by publications covering only infectious diseases.

Fungal Diseases			
Disease Name	Aetiological Agent	Number of Publications	Zoonotic Potential
Epizootic lymphangitis	*Histoplasma capsulatum* variety *farciminosum*	7	No
Aspergillosis	*Aspergillus* spp.	1	No
Histoplasmosis	*Histoplasma* spp.	1	No
Penicillosis	*Penicillum* spp.	1	No
Candidosis	*Candida* spp.	1	No
Cryptococcosis	*Cryptococcus* spp.	1	No
Geotrichosis	*Geotrichum* spp.	1	No
Trichophytosis	*Trichophyton* spp.	1	Yes
Microsporosis	*Microsporum* spp.	1	Yes

**Table 8:** Infectious diseases reported in articles covering both infectious and non-infectious diseases.

Infectious Diseases Within Records Covering Multiple Diseases			
Disease Name	Aetiological Agent	Type of Aetiologicl Agent	Zoonotic Potential
African horse sickness	*Orbivirus* genus	Virus	No
Rabies	*Lyssavirus* genus	Virus	Yes
Anthrax	*Bacillus* anthracis	Bacterium	Yes
Ulcerative lymphangitis	*Corynebacterium pseudotuberculosis*	Bacterium	Yes
Strangles	*Streptococcus equi*	Bacterium	Some subspecies only
Tetanus	*Clostridium tetani*	Bacterium	No
Epizootic lymphangitis	*Histoplasma capsulatum* variety *farciminosum*	Fungus	No
Gastrointestinal parasites	Unspecified	Helminth	Unspecified
Mites	Unspecified	Ectoparasite	Unspecified

**Table 9:** Effects on sick animals, economic and social impacts of individual diseases or multifactorial conditions or diseases determined by the same group of pathogens described within eligible publications.

Disease	Effects on Affected Animals	Economic Impacts on Owners	Social Impacts on Owners
Epizootic lymphangitis	Animal death; reduced work productivity; reduced load capacity; reduced work time; incapacity to work during last disease stages; bad animal smell; poor animal appearance	Reduced income; animal loss; need for animal replacement; high expenses for ineffective treatment; loss of clients; unemployment; damaged livelihood; poverty	Stigmatisation; loss of motivation; mental health problems; poverty
African horse sickness	High mortality rates	Animal loss; loss of breeding potential; loss of previous investments in animal care	Mental health problems
Equine trypanosomiasis	Death; reduced animal efficiency	Reduced income; animal loss in absence of treatment; high treatment costs	Not reported
Equine infectious anaemia	Reduced animal efficiency	Travel bans of infected animals; loss of animal economic value	Not reported
Gastrointestinal helminthiasis	Reduced animal efficiency	Reduced income; high treatment costs	Not reported
Lameness and foot diseases	Inability to work for long periods	Reduced income; loss of animal economic value; high treatment costs	Not reported

## Data Availability

Not applicable.

## Supplementary Materials

The following supporting information can be downloaded at: https://www.mdpi.com/article/10.3390/ani13243865/s1, Supplementary Material S1: Search strategies applied to the various databases including the World Bank filter (Cochrane, 2020). Supplementary Material S2: Reference list of publications included in the review.
