# Supplementary material for: The Socioeconomic Impact of Diseases of Working Equids in Low and Middle-Income Countries: A Critical Review
Source: Animals (Basel). 2023 Dec 15;13(24):3865. doi: 10.3390/ani13243865 (PMC10741040; doi:10.3390/ani13243865)
Supplement: Supplementary file 1 [file animals-13-03865-s001.zip › animals-2679331-supplementary/Supplementary material S2.pdf]

Supplementary material 2 – Reference list of publications included in the review

Eligible publications retrieved from the database search

Ali, A.; Orion, S.; Tesfaye, T.; Zambriski, J.A. The prevalence of lameness and associated risk factors in cart mules in Bahir Dar, Ethiopia. *Trop. Anim. Health Prod.* 2016, 48, pp. 1483-1489. doi: 10.1007/s11250-016-1121-7

Bekele, M.; Leggese, G.; Teshome, W.; Nahom, W.; Anteneh, K.; Tewodros, T. Socioeconomic impact of epizootic lymphangi-tis in cart mules in Bahir Dar city, North-West Ethiopia. In *How do we demonstrate the importance of working equid welfare to human livelihoods?,* Proceedings of the 7th International Colloquium on Working Equids, Royal Holloway, University of Lon-don, United Kingdom, 1st - 3rd July 2014; World Horse Welfare: Snetterton, United Kingdom, 2014; pp. 10-15

Duguma, B.E.; Tesfaye, T.; Kassaye, A.; Kassa, A.; Blakeway, S.J. Control and Prevention of Epizootic Lymphangitis in Mules: An Integrated Community-Based Intervention, Bahir Dar, Ethiopia. *Front. Vet. Sci.* 2021, 8, pp. 1-18. doi: 10.3389/fvets.2021.648267

Etana, D. Isolates of fungi from symptomatic carthorses in Awassa, Ethiopia. *J. Vet. Med B. Infect. Dis. Vet. Public Health* 1999, 46(7), pp. 443-451. doi: 10.1046/j.1439-0450.1999.00251.x

Gichure, M.; Onono, J.; Wahome, R.; Gathura, P. Analysis of the benefits and production challenges of working donkeys in smallholder farming systems in Kenya. *Vet. World* 2020, 13(11), pp. 2346-2352. doi: 10.14202/vetworld.2020.2346-2352

Kumar, R.; Jain, S.; Kumar, S.; Sethi, K.; Kumar, S.; Tripathi, B.N. Impact estimation of animal trypanosomiasis (surra) on livestock productivity in India using simulation model: Current and future perspective. *Vet. Parasitol. Reg. Stud.* 2017, 10, pp. 1-12. doi: 10.1016/j.vprsr.2017.06.008

Molla, A.M.; Fentahun, T.; Jemberu, W.T. Estimating the Economic Impact and Assessing Owners' Knowledge and Practices of Epizootic Lymphangitis in Equine Cart Animals in Central and South Gondar Zones, Amhara Region, Ethiopia. *Front. Vet. Sci.* 2021, 8, pp. 1-10. doi: 10.3389/fvets.2021.673442

Nigatu, A.; Abebaw, Z. Socioeconomic impact of Epizootic Lymphangitis (EL) on horse-drawn taxi business in Central Ethi-opia. In *Learning from others*. Proceedings of an International

Colloquium, New Delhi, India, 29 November - 2 December 2010, The 6th International Colloquium on Working Equids, New Delhi, India, 29 November – 2 December 2010; The Brooke: London, 2010; pp. 83-86

Nogueira, M.F.; Oliveira, J.M.; Santos, C.J.S.; Petzold, H.V.; Aguiar, D.M.; Juliano, R.S. et al. Equine infectious anaemia in equids of Southern Pantanal, Brazil: seroprevalence and evaluation of the adoption of a control programme. *Pesqui. Vet. Bras.* 2017, 37(3), pp. 227-233. doi: 10.1590/s0100-736x2017000300005

Redmond, E. F.; Jones, D.; Rushton, J. Economic assessment of African horse sickness vaccine impact. *Equine Vet. J.* 2021, 54, pp. 368-378. doi: 10.1111/evj.13430

Scantlebury, C.E.; Pinchbeck, G.L.; Loughnane, P.; Aklilu, N.; Ashine, T.; Stringer, A.P. et al. Participatory appraisal of the impact of epizootic lymphangitis in Ethiopia. *Prev. Vet. Med.* 2015, 120, pp. 265-276. doi: 10.1016/j.prevetmed.2015.03.012

Solomon, A.; Fekadu, A.; Molla, B.; Sheferaw, D. The prevalence of foot related problems in working donkeys and its implication on the livelihood of donkey owners in Hawassa City, Southern Ethiopia. *Int. J. Livest. Prod.* 2019, 10(3), pp. 86-93. doi: 10.5897/IJLP2018.0527

Relevant publications obtained by screening the references of the eligible articles

Admassu, B.; Shiferaw, Y. Donkeys, horses and mules – their contribution to people's livelihoods in Ethiopia. Addis Ababa: The Brooke. 2011. Available online: https://www.thebrooke.org/sites/default/files/Ethiopia-livelihoods-2020-01.pdf (accessed on 15th April 2022)

Angara, T.E.E.; Ismail, A.A.; Ibrahim, A.M. The role of donkeys in income generation and the impact of endoparasites on their performance. *University of Khartoum Veterinary Journal of Veterinary Medicine and Animal Production* 2011, 2(2), pp. 65-89. Available online: https://www.researchgate.net/publication/300368804_THE_ROLE_OF_DONKEYS_IN_INCOME_GENERATION_AND_THE_IMPACT_OF_ENDOPARASITES_ON_THEIR_PERFORMANCE (accessed on 18 April 2022)

Grewar, J.D.; Weyer, C.T.; Guthrie, A.J.; Koen, P.; Davey, S.; Quan, M. et al. The 2011 outbreak of African horse sickness in the African horse sickness controlled area in South Africa. *J. S. Afr. Vet. Assoc.* 2013, 84(1), pp. 1-7. doi: 10.4102/jsava.v84i1.973

Mitku, M.; Assefa, A.; Abrhaley, A. Prevalence, associated risk factors and socioeconomic impact of Epizootic lymphangitis (EL) in carthorses in and around Gondar town. *J. Am. Sci.* 2018, 14(2), pp. 77-83. doi: 10.7537/marsjas140218.10

Seidl, A.; Moraes, A.S.; Silva, R.A.M.S. A financial analysis of treatment strategies for *Trypanosoma evansi* in the Brazilian Pantanal. *Prev. Vet. Med.* 1998, 33, pp. 219-234. doi: 10.1016/S0167-5877(97)00049-4

Other eligible articles sourced from the references of the five above listed relevant publications

Jagema, T.; Jarso, D. Study on Epidemiology and Socioeconomic Impact of Epizootic Lymphangitis in Carthorses in Southwestern Shoa. *Open Access J. Vet. Sci. Res.* 2016, 1(3), pp. 1-10. Available online: https://medwinpublishers.com/OAJVSR/OAJVSR16000114.pdf (accessed on 16 April 2022)

List of relevant publications obtained by screening the index of whole conference proceedings and special issues retrieved from the database search

Proceedings

Valette, D.; Upjohn, M. Voices from women: working equids as 'invisible helpers'. In *How do we demonstrate the importance of working equid welfare to human livelihoods?* Proceedings of the 7th International Colloquium on Working Equids, Royal Hol-loway, University of London, United Kingdom, 1st-3rd July, 2014; World Horse Welfare: Snetterton, United Kingdom, 2014; pp. 16-18

Special issues

Martin Curran, M.; Feseha, G.; Smith, D.G. The Impact of Access to Animal Health Services on Donkey Health and Livelihoods in Ethiopia. *Trop. Anim. Health Prod.* 2005, 37(1), pp. 47-65. Available online: https://link-springer-com.ezproxy.is.ed.ac.uk/journal/11250/volumes-and-issues/37-1/supplement (accessed on 19 March 2022)
